# Application of Gelatin for Sustainable Stabilization of Low-Compressible Silt–Clay Mixtures: Geotechnical Behavior and Carbon Emission Considerations

**DOI:** 10.3390/polym17141954

**Published:** 2025-07-17

**Authors:** Evangelin Ramani Sujatha, Veera Ragavan Pratheeba, Jair De Jesus Arrieta Baldovino, Yamid E. Nunez de la Rosa

**Affiliations:** 1 Centre for Advanced Research in Environment, School of Civil Engineering, SASTRA Deemed University, Thanjavur 613401, India; vpradeepa95@gmail.com; 2 Applied Geotechnical Research Group, Civil Engineering Program, Universidad de Cartagena, Cartegena de Indias 130015, Colombia; jarrietab2@unicartagena.edu.co; 3 Faculty of Engineering and Basic Sciences, Fundacion Universitaria Libertadores, Bogota 111221, Colombia; yenunezd@libertadores.edu.co

**Keywords:** biopolymer, gelatin, compaction behavior, deformation behavior, UCS, carbon footprint

## Abstract

Biopolymers, owing to their environmentally friendly and sustainable characteristics, have become a promising alternative for soil stabilization in geotechnical engineering. The application of protein-based biopolymers as binders for soil stabilization is less prevalent in geotechnical engineering compared to polysaccharide-based biopolymers. This study explores the potential of gelatin, a protein-based biopolymer derived from animal collagen, for stabilizing silty sand and improving its geotechnical properties. Gelatin was mixed into the soil at concentrations ranging from 0.25% to 2% of the dry weight of soil, and its effects on various soil characteristics were evaluated. The tests conducted include liquid limit, plastic limit, compaction behavior, and unconfined compressive strength (UCS); the addition of 1% gelatin led to an approximate 1.69 times increase in the strength of the unamended soil. After 28 days of curing, the UCS improved by approximately 5.03 times compared to the untreated soil, and the treated soil exhibited increased resistance to deformation under load. Microstructural analysis using scanning electron microscopy (SEM) revealed that gelatin facilitated the formation of a cohesive matrix, enhancing particle bonding and reducing void spaces within the soil. Carbon footprint analysis (CFA) conducted on an isolated footing stabilized with gelatin showed that the carbon emissions were reduced by 99.8% and 99% compared to traditional stabilizers such as lime and cement. Additionally, the interaction between the biopolymer and the fine-grained soil is distinctly evident in the FTIR and XRD analysis through hydrogen bonding and the formation of cementitious compounds.

## 1. Introduction

Soil stabilization is a crucial process in construction and engineering projects to improve the strength and durability of soil. The stabilization techniques include mechanical stabilization, chemical stabilization, and stabilization using additives. Mechanical stabilization involves mixing different soil gradations to achieve the desired specifications [1]. Chemical stabilization is an effective and commonly used method for enhancing soil properties. This technique involves inducing chemical reactions between soil minerals and stabilizing agents like cement and lime, and thereby stabilized soil materials with greater strength are produced using this technique [2]. However, growing concerns regarding environmental pollution and greenhouse gas emissions have raised significant questions about the use of cement and lime, as their production processes are major sources of carbon dioxide emissions globally [3]. The production of cement is expected to grow from 2.5 billion tons in 2016 to 4.4 billion tons by 2050, marking a 23.8% increase and responsible for approximately 8% of global CO_2_ emissions [4]. Researchers have introduced sustainable solutions, such as biopolymers and biological techniques like microbially induced calcite precipitation (MICP) [5]. The MICP technique involves using bacteria to break down urea, generating carbonate ions that react with a calcium-rich solution to bind soil particles, thereby improving soil strength and stiffness [6]. However, MICP has several limitations in field implementation. While it is most effective for coarse-grained soils, it encounters challenges when applied to fine-grained soils due to infiltration issues. Also, pore sizes in fine-grained sediments create an unsuitable environment for bacterial growth, and the production of ammonium as a byproduct further complicates the process [7]. Many researchers have therefore concentrated on directly utilizing biogenic excrement (i.e., biopolymers) rather than trying to cultivate microorganisms in the soil. Biopolymers are natural polymers that are synthesized naturally in the environment by plants and living organisms shown exceptional potential in enhancing soil properties for geotechnical engineering, particularly their strength [8]. The three primary types of biopolymers commonly considered are polynucleotides (such as RNA and DNA), polypeptides (composed of amino acids), and polysaccharides, which are the most frequently used in various engineering applications [9]. Biopolymers like xanthan gum, guar gum, gellan gum, chitosan, sodium alginate, casein, carrageenan, gelatin, and β-glucan have been examined for their potential to modify the soil properties. The incorporation of xanthan gum into the clay led to a substantial increase in compressive strength, which increased by approximately 470% [10]. Gellan gum also demonstrates considerable soil strengthening properties, with the UCS of 3% gellan gum-treated sandy lean clay (CL) reaching as high as 12.6 MPa [11]. β-glucan biopolymer shows significant strengthening in sandy lean clay (CL), with 0.25% and 0.5% content having UCS values of 2.17 MPa and 4.31 MPa, respectively, both higher than the UCS of 10% cement-treated condition (2.65 MPa) [12]. Chitosan showed a remarkable 103% increase in strength after 28 days of curing and exhibited no signs of degradation, highlighting its potential as a durable and reliable soil stabilizer [13]. Sodium alginate and agar significantly enhanced soil compressive strength, achieving over 85% of the maximum strength with just 0.5% addition by weight [14]. The addition of nano carboxymethyl cellulose (nCMC) led to the UCS increasing by 1.68 times after 90 days of curing [15]. Casein, a protein-based biopolymer identified as an effective soil binder, significantly enhancing strength. For instance, a 6.66% casein addition can increase the UCS of sandy soils by up to 5.63 MPa, the polymeric chains of casein penetrate the soil particles, binding the soil and biopolymer together through electrostatic interactions between the protein functional groups [16]. The interaction between the zein biopolymer (a protein-based biopolymer) and fines helped to bridge the coarse particles, improving the bond between the fine and coarse particles at higher biopolymer concentrations [17]. Gelatin was also used to stabilize different types of soil, including high compressible clay, low-compressible clay, and low-compressible silt, to enhance their mechanical properties, such as California Bearing Ratio (CBR) and strength [18].

Polysaccharide biopolymers like xanthan gum, guar gum, gellan gum, etc. have been investigated extensively for their potential to improve soil properties. This study explores the use of gelatin, a protein-based biopolymer, to improve the geotechnical properties of silty soil. Protein-based biopolymers are more resistant to degradation, especially in wet conditions, show stronger cross-linking with soil properties, and provide long-term stability [19]. Gelatin, which has not been extensively studied for soil stabilization, is evaluated for its potential in enhancing various geotechnical properties, including unconfined compressive strength, compaction behavior, viscosity, pH, and deformation characteristics. The study involves tests such as the Unconfined Compressive Test to assess strength improvements, FTIR and SEM to analyze the chemical interactions and microstructural changes between gelatin and soil, XRD to evaluate any changes in the crystalline structure, viscosity to examine the thickening effect of gelatin, and pH to monitor any changes in soil acidity. Various dosages of gelatin, ranging from 0.25% to 2% of the dry weight of soil, are tested to determine the optimal concentration for soil stabilization. The results suggest that gelatin significantly enhances the strength and stability of silty soil, making it a promising material for applications such as foundation stabilization and slope stabilization.

## 2. Materials and Methods

### 2.1. Soil

**Figure 1:**
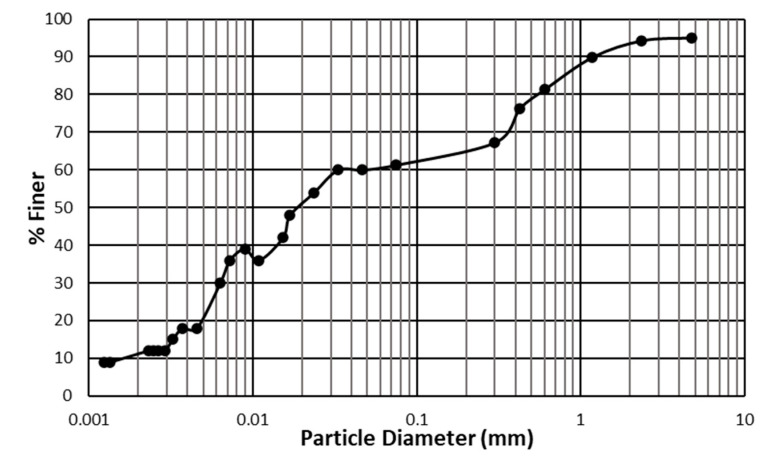
Gradation curve of the selected soil.

Soil was extracted from trenches 2 m deep from Thiruvaiyaru town of Thanjavur district in Tamil Nadu. The trenches were exclusively made for soil sample collection to avoid the topsoil and organic matter. The soil was brown and had a specific gravity of 2.71. The organic content in the soil is less than 1%. The soil has nearly 60% fines content and an effective size of 0.0017 mm. The gradation curve is shown in Figure 1.

The select geotechnical properties of the soil are listed in Table 1. The soil is low-plastic and is classified as a low-compressible silt–clay mixture (ML-CL) according to unified soil classification. The soil contains 38% sand, 50% silt, and 12% clay. The soil attains a high unit weight at a low moisture content, as observed from Table 1. The UCS of the soil is 121.91 kPa, indicating that the soil is in stiff consistency at its OMC.

### 2.2. Gelatin

**Figure 2:**
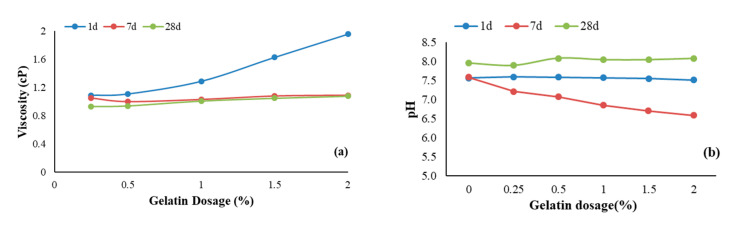
Change with dosage and time in (**a**) viscosity and (**b**) pH.

Gelatin is a biopolymer usually derived from animal protein obtained by partial hydrolysis of collagen, which can be extracted from sources like animal bone, connective tissue, skin, etc. Gelatin was procured from Neoteric DCBA Ideas, Coimbatore, Tamil Nadu, India. It is pale yellow and is soluble in hot water. Gelatin is used in cosmetics and the health-care industry owing to its excellent gel-forming property (Alipal et al. 2019) [20]. It forms thermoreversible gels which dissolve on heating. Gelatin forms both gel-plug and biofilm in 2 h. The pH of the gelatin-stabilized soil ranges from 6.7 to 8.08. The pH of gelatin-stabilized soil is lower on 7 d and increases to a slightly alkaline range of 8 at 28 d. The drop in pH from 1 d to 7 d can be due to consumption of the alkaline species like Ca^2+^, OH^−^ in the soil to hydrates like CSH, and this breakdown may be due to partial hydrolysis, as gelatin is organic. Further increase in pH from 7 d to 28 d, recovering near the initial level, may be due to calcium carbonate precipitation after carbonation and hydration reactions, which can be verified from XRD analysis. Gelatin is hydrophilic [20]. The viscosity of gelatin varies with both dosage, temperature, and ageing. In this study, the change in viscosity with dosage and ageing is investigated. The viscosity of gelatin is presented in Figure 2. It can be observed from Figure 2a that the viscosity increases with dosage but decreases with ageing, and over time, the rate of change in viscosity is notably minimal with an increase in dosage. The loss of soluble protein in the fluid phase would have led to a decrease in viscosity [21].

## 3. Experimental Investigation

Soil was cleaned to remove any undesirable matter and was then oven dried at 100 °C for 24 h in a thermostatically controlled oven to remove any natural water content. The soil was then sieved through appropriate sieves for various experimental investigations. Gelatin dosages of 0.25, 0.5%, 1.0%, 1.5% and 2.0% of the dry weight of soil were selected for the study. Soil and gelatin were mixed using a wet mixing procedure, i.e., gelatin was mixed with water, and this was then gradually added to the soil and was thoroughly hand-mixed to attain a uniform distribution within the soil matrix. The soil–gelatin mixtures were allowed to rest for a period of 3 h before all the tests in airtight covers. UCS samples were molded at the OMC into cylinders of 38 mm diameter and 76 mm height.

Liquid limit and plastic limit tests were conducted in line with the procedures given in ASTM D4318 [22]. Casagrande’s cup was used to determine the liquid limit and thread rolling for the plastic limit. The plasticity index is calculated as the difference between the liquid and plastic limit. Light compaction effort was imparted to determine the OMC and MDUW following the guidelines outlined by [8]. UCS test was carried out after curing for 1 d, 7 d, and 28 d at the above-mentioned dosages to study the effect of gelatin addition on strength during short-term ageing. The soil–gelatin mixtures were allowed to rest for 3 h to attain equilibrium moisture content, then cylinders were molded of the mentioned sizes, and these cylinders were air-cured for the remaining curing period. The UCS test was conducted in accordance with ASTM D2166 [23]. A minimum of three trials were conducted for all the dosages in every geotechnical parameter investigated. The average of the results is presented, and the percentage variation between the trials was less than 5%. Bruker XRD equipment was used for X-ray diffraction analysis, and micrographs to interpret the changes in surface morphology were obtained from a TESCAN Vega 3 scanning electron microscope (SEM). Similarly, Fourier Transform Infrared (FTIR) spectroscopy was conducted using a PerkinElmer spectrometer (Waltham, MA, USA) to investigate the interaction between gelatin and soil particles. The spectra were recorded in the range of 4000–400 cm^−1^.

## 4. Results and Discussion

### 4.1. Liquid and Plastic Limit

**Figure 3:**
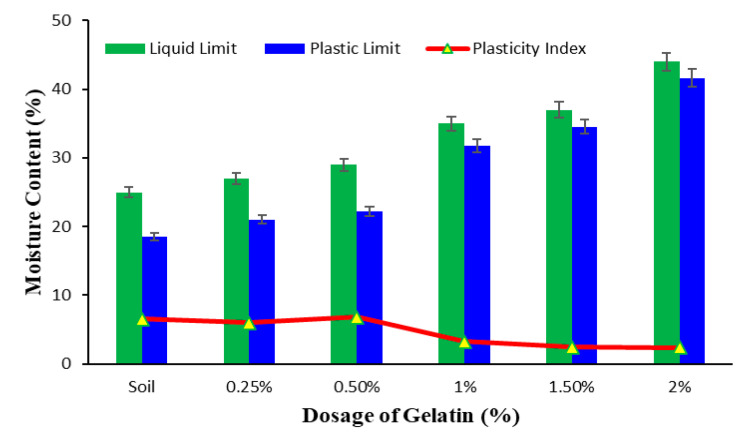
Effect of gelatin dosage on the plastic behavior of soil.

**Figure 4:**
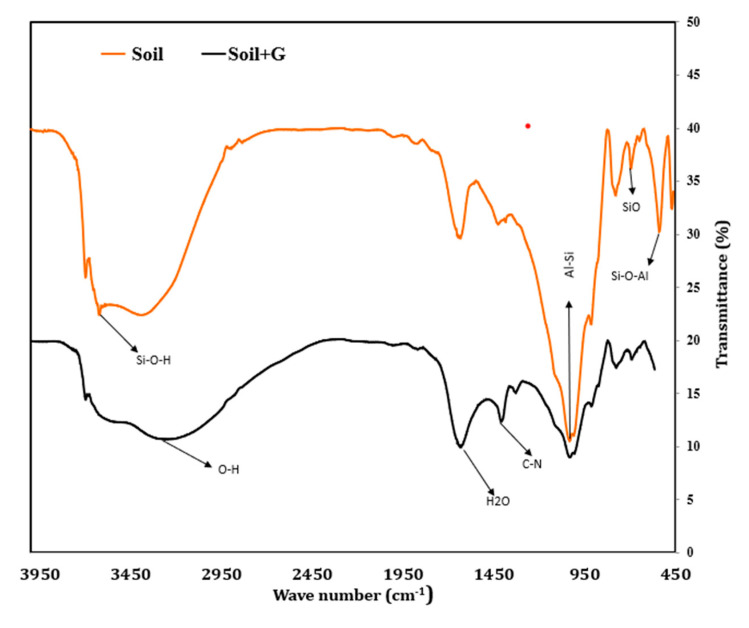
FTIR spectra of soil and gelatin-stabilized soil.

Liquid limit, plastic limit, and plasticity index of the soil are vital tools for predicting soil behavior. Gelatin increases both the liquid and plastic limit of the soil (Figure 3). The liquid limit increases from 25% for unstabilized soil to 44% for soil stabilized with 2% gelatin, exhibiting an increase of 76%. The plastic limit shows an increase of nearly 1.28 times with the addition of 2% gelation in comparison to the soil. The hydrophilic nature of gelatin increases water absorption and retention, leading to an increase in both the liquid and plastic limits. This is evident from the FTIR spectra of the stabilized soil (Figure 4). Gelatin contains amino acid and hydroxyl groups that retain and hold water. The hydroxyl groups add to existing –OH stretching from soil to adsorbed water and are observed in the FTIR spectra of stabilized soil in the form of broader and intense absorption in this region (Figure 4), indicating water absorption, which could have contributed to an increase in liquid limit.

The FTIR spectra of stabilized soil also show new amide bands, and this indicates that gelatin interacts with the soil surface by coating the soil particles and forming gel-like structures. These new gel structures in the soil matrix increase the water content required to make the stabilized soil matrix flow. Also, these gel formations bind the soil particles together, increasing the water content necessary for plastic behavior, causing an increase in the plastic limit of the stabilized soil.

The rate of increase in plastic limit is notably higher than that of liquid limit, leading to a decrease in plasticity index with the increase in gelatin dosage. The plasticity index falls by 65% on the addition of 2% gelatin compared to that of the soil. This decrease in plasticity index indicates that the soil is less plastic over a range of water content. The gelatin-stabilized soil, therefore, has reduced workability but improved stability. The reduced plasticity index can be attributed to the tendency of gelatin to bind soil particles, deterring its capacity to flow, limiting its liquid limit, while the same binding increases the ability to retain water, causing an increase in plastic limit. The presence of new amide peaks in the FTIR spectra (Figure 4) of stabilized soil indicates the inclusion of gelatin’s protein structure, which in turn promotes interparticle bonds, leading to higher plastic limit and lesser plasticity index. The reduced plasticity index makes this gelatin-stabilized soil more suitable for engineering applications where strength enhancement is the primary criterion, alongside slope stabilization, erosion control, and subgrade and foundation bed stabilization. However, at the same time, this is not suitable for applications of clay liners. The soil type must also be taken into account when exploring the possibility of using gelatin as a soil stabilizer.

### 4.2. Compaction Behavior

**Figure 5:**
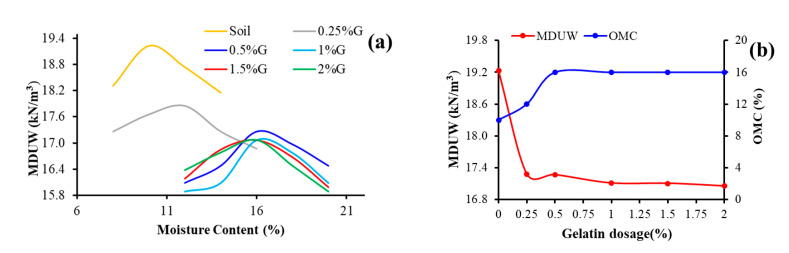
Compaction behavior of gelatin-stabilized soil. (**a**) Effect of Gelatin dosage on MDUW and OMC; (**b**) Comparison of MDUW and OMC at varying Gelatin dosage.

The compaction behavior of the gelatin-stabilized soil is significantly different from that of soil. The compaction curves show a notable shift downward and toward the right side, indicating lower MDUW and higher OMC (Figure 5a). The shape of the compaction curves remains concave downward, distinctly with a clear peak, indicating that the stabilized soil is sensitive to changes in water content. The gelatin dosage being minimum, the compaction curves show minimal difference in MDUW with respect to dosage, but there is a discernable shift to the right due to a notable increase in OMC. The OMC increased with gelatin addition at lower dosages, but at higher dosages beyond 1% remained the same. This can indicate that at 1% gelatin, a saturation point would have been reached at which gelatin hydrogel’s water holding capacity reaches the maximum, and further addition does not impact water addition proportionally. The change in OMC is significant, increases by 20% at 0.5% gelatin addition and 60% for further investigated higher gelatin dosages (Figure 5b). The hydrophilic nature of gelatin leads to a significant increase in OMC. The MDUW decreased with an increase in gelatin dosage minimally. The MDUW showed a reduction of nearly 11.3% at the maximum investigated dosage of 2% gelatin (Figure 5b). The decrease in MDUW can be due to the resistance offered to closer particle packing by the gel network formed on the addition of gelatin. The gel network increases interparticle bonding by holding soil particles together, but resists closer packing of the soil particles. The gel (hydrogel) swells with water addition, increasing the volume of the soil–gelatin matrix, which again leads to a decrease in MDUW. Also, gelatin is lighter in weight compared to soil and can trap air or water, which also may resist soil particle packing, leading to a marginally reduced MDUW.

### 4.3. Deformation Behavior

**Figure 6:**
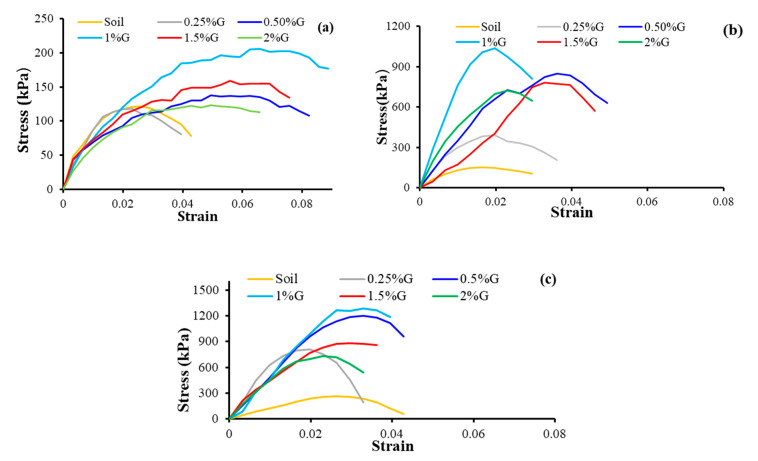
Stress–strain behavior (**a**) 1 d curing; (**b**) 7 d curing, and (**c**) 28 d curing.

The deformation behavior of gelatin-stabilized soil is impacted by both dosage and ageing, as observed from Figure 6a–c. The curves exhibit an initial linear region at lower stress levels, which is more pronounced with ageing, owing to the tendency of gelatin to bind soil particles, effectively increasing the cohesion in the stabilized soil matrix. The stabilized soil exhibits a gradual reduction in post-peak strength during the early curing period of 1 d, but with ageing, there is a significant change after peak behavior. There is a notable reduction in post-peak strength as the curing period increases, indicating that the stabilized soil becomes brittle with ageing. The strain also shows a marginal decrease, indicating that the stabilized soil has increased in stiffness and strength, making it more suitable for geotechnical applications like pavement and foundation beds. During the early curing period, the hydrogels allow plastic deformation owing to their flexible nature and also redistribute stress through the stabilized soil matrix, which leads to a gradual increase in strain, but with ageing, the hydrogels become stiffer on dehydration and resist higher loads at lower strains, showing strain hardening behavior. The stabilized soil exhibits a pronounced increase in stress at 1% gelatin addition. Gelatin coats the soil matrix, enhancing aggregation, flocculation, and bridging, which causes the stabilized soil to show a stiffer stress–strain response. With ageing, the hydration reactions yield more cementitious products, and hence, with ageing, the stabilized soil is brittle as observed from its stress–strain response (Figure 6b,c).

Gelatin promotes minor cementitious reactions that lead to the formation of cementitious products like calcium silicate hydrates (CSH) and calcium carbonate (CaCO_3_) in the gelatin-stabilized soil, as observed from the XRD spectrum, as minor peaks that bind particles together, increasing their resistance to loads. The drop in pH (Figure 2b) of gelatin-stabilized soil is an indicator of carbonation and formation of CaCO_3_, and this leads to the stiffness of the stabilized soil matrix.

**Figure 7:**
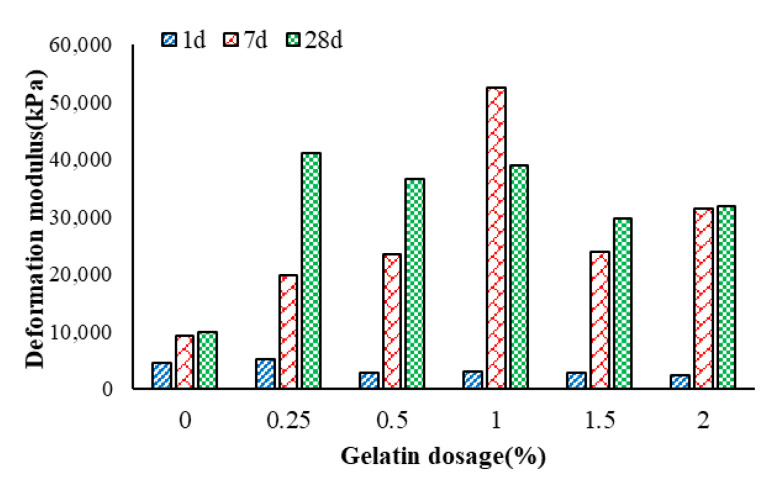
Effect of gelatin on the deformation modulus of the soil.

The deformation modulus (ratio of stress at failure to corresponding strain) increases with an increase in gelatin dosage and ageing (short-term), as observed from Figure 7. At all investigated dosages and curing periods, the deformation modulus is higher than that of soil due to optimal cross-linking and soil particle bridging, early formation of CSH, and greater particle bonding due to carbonation that leads to the formation of CaCO_3_ that fills the voids in the soil matrix [21]. After the 7d curing period, there is an abnormal increase in deformation modulus after 1% gelatin addition, and this can be attributed to the optimum gelatin dosage to effectively bind soil particles and promote hydration/carbonation without the excess gelatin that can soften the soil matrix. However, at 28 d, pH rises again, and other dosages again favorable environment, almost equivalent to that at 7 d for 1%.

### 4.4. UCS

**Figure 8:**
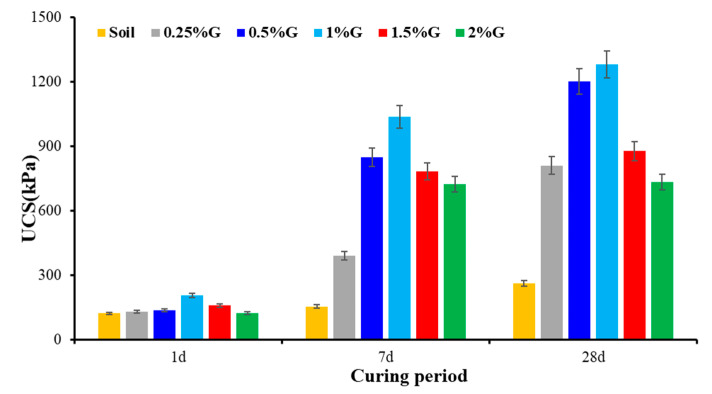
Strength gain with gelatin addition at the investigated dosages.

UCS of gelatin-stabilized soil is higher than the unstabilized soil at all investigated dosages and curing periods (Figure 8). The results show that 1% gelatin yielded the highest increase in strength at all curing periods and hence is the optimum dosage for this investigated soil, a low-compressible silt–clay mixture. At the minimum dosage of 0.25%, the UCS increased marginally by 6.47% at 1 d curing and significantly by nearly 209% after 28 d curing. At the optimum dosage of 1%, the increase in UCS at 1 d and 28 d curing was around 69% and 389%, respectively. At the highest investigated dosage of 2%, the increase in strength was minimum at around 1% at 1 d curing and 64.33% after 28 d curing. The results show that gelatin-stabilized soil exhibits increased strength with ageing, which makes it suitable for long-term applications. The increase in strength can be attributed to the formation of hydrogels and hydrogen bonds. Beyond 1% dosage, excess gelatin begins to accumulate in the pore spaces and may act as a soft, lubricating medium rather than a reinforcing phase. This reduces effective stress transfer between particles, leading to a decrease in shear strength.

**Figure 9:**
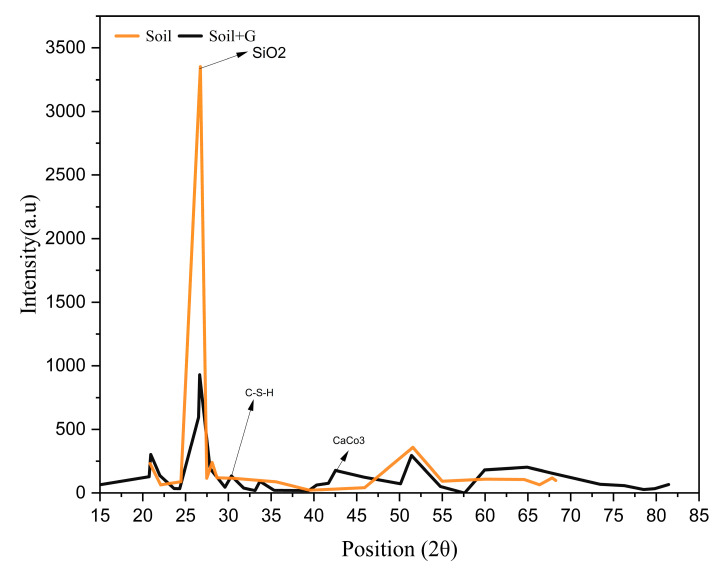
XRD diffractogram for soil and gelatin-stabilized soil.

The FTIR spectra exhibit stretching bands around 3224 cm^−1^ and 1616 cm^−1^ corresponding to OH stretching, indicating water adsorption and the formation of hydrogen bonds between the soil particles and gelatin, which contributes to greater binding and increased resistance to deformation. This interaction is primarily attributed to the presence of amide groups in gelatin, which facilitate hydrogen bonding and contribute to enhanced interparticle binding and increased resistance to deformation as seen from Figure 4 [24]. The XRD spectrum (Figure 9) shows the presence of hydration products like CSH, which would have contributed to the increase in UCS.

**Figure 10:**
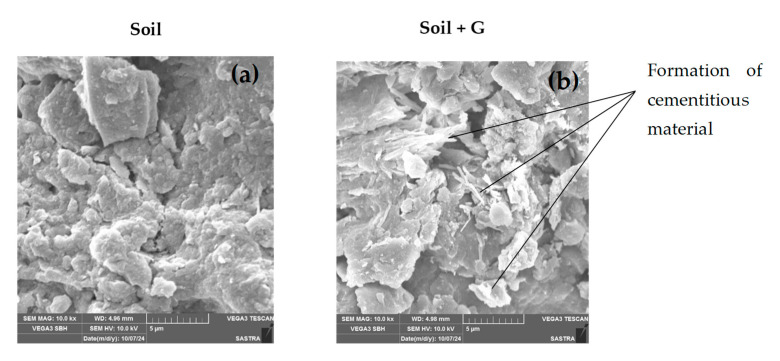
Micrographs of (**a**) soil and (**b**) soil stabilized with gelatin.

Gelatin acts as a binder, facilitating the reaction between the calcium and silica present in soil, leading to the formation of CSH (Figure 9). The XRD spectra also show a decreased intensity of quartz present in the soil matrix, which may be due to its consumption in pozzolanic reaction, leading to the formation of CSH. The CaCO_3_ crystals, as observed from the XRD spectrum presented in Figure 9 [25] and visualized in SEM micrographs (Figure 10), nucleate in pore spaces, filling the voids, rendering the stabilized soil matrix denser, which in turn may contribute to the increase in UCS [26].

The micrographs from SEM (Figure 10) also show the presence of crystalline formations that may have contributed to the increased strength and stiffness of the gelatin–soil matrix. The mechanism of strength gain can be summarized by the following biochemical equations [27]

Hydrolysis: Gelatin + Water → Peptides + Amino Acids

CSH formation: **SiO_2_ + Ca(OH)_2_ + H_2_O → CSH** (increases cohesion and interparticle bonding)

Carbonation: **Ca(OH)_2_ + CO_2_ → CaCO_3_ + H_2_O** (imparts strength gain over time)

### 4.5. Mechanism of Improvement

The soil is a low-compressible silt–clay mixture. Gelatin is a protein-based biopolymer that forms a gel when added to water. The hydrophilic protein chains (amino acids and hydroxyl groups, as observed from the FTIR spectra in Figure 4) in gelatin interact with the soil particles. The hydrophilic nature enhances water absorption and retention, leading to an increase in liquid, plastic limit, and OMC. Gelatin also forms minor cementitious phases, CSH and CaCO_3_, as observed from the XRD spectra (Figure 9). Figure 2b shows that the pH at 1 d remains between 7.5 and 7.6 at all investigated dosages, indicating minimum reaction between gelatin and soil. At 7 d, the pH drops notably, and this can be attributed to a combination of gelatin hydrolysis, formation of CSH, and carbonation. At 28 d, the pH increases to a range between 7.7 and 8.1, which can be owing to the completion of CSH formation and conversion of bicarbonates formed during carbonation to CaCO_3_.

**Figure 11:**
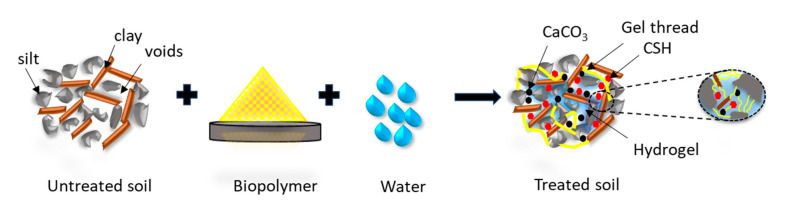
Schematic sketch of the improvement mechanism on gelatin addition.

Gelatin forms gel with water and coats the soil particles, which, along with the cementitious products, aggregates soil particles and promotes flocculation. The hydrogel networks resist closer packing of the soil particles, which leads to a marginal reduction in MDUW. The flocculation and cementation in stabilized soil may also be attributed to a decrease in the plasticity index. The cementitious products and gelatin gel fill the voids in the soil matrix, rendering the soil matrix denser. The SEM micrographs (Figure 10) show coating and cementitious products with reduced void spaces compared to unstabilized soil, which exhibits discrete plate-like particles with visible void spaces. The stabilized matrix with lesser voids and cementitious products improves the stiffness of the stabilized soil matrix and imparts a brittle nature, particularly on ageing, as with ageing, the hydrogels harden on dehydration with higher strength. The combination of coating, gel network, and cementitious products improves the interparticle bonding in the stabilized soil matrix, yielding higher UCS. The mechanism of change in the stabilized soil matrix is presented in Figure 11. The viscosity increases with dosage (Figure 2a), and this correlates with higher water retention, leading to an increase in liquid limit, plastic limit, and OMC with dosage. It also indicates enhanced cohesion as reflected in the UCS (Figure 8).

### 4.6. Carbon Footprint Analysis of Gelatin-Stabilized Footing

**Figure 12:**
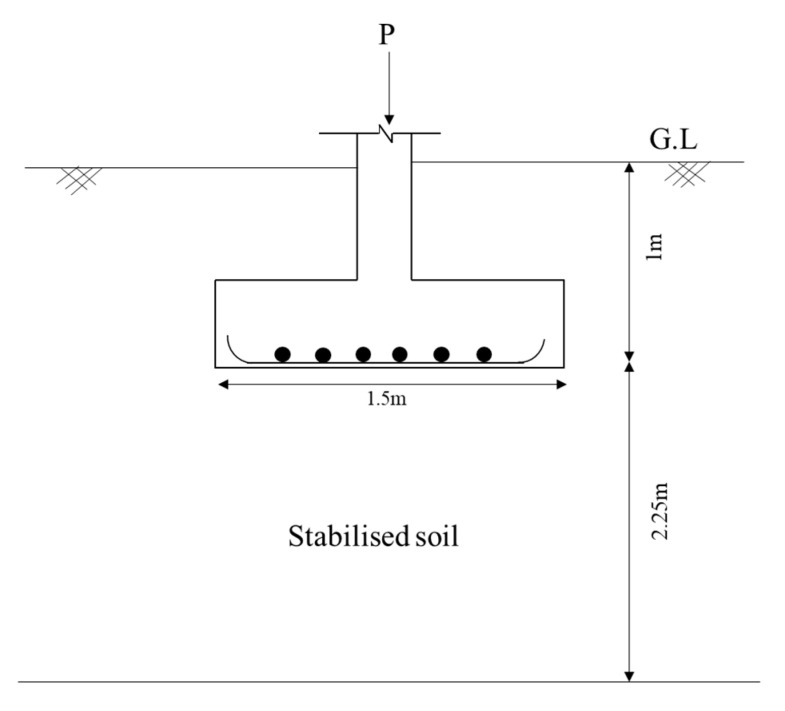
Typical cross-section of an isolated footing.

Carbon footprint analysis is the process of measuring the total greenhouse gas (GHG) emissions produced by a product, material, or activity throughout its lifecycle. It considers emissions from raw material extraction, manufacturing, transportation, usage, and disposal. This analysis helps identify environmental impacts and supports sustainable decision-making to reduce carbon emissions [28]. This work involved the evaluation of CO_2_ emissions at different stages of footing construction. A typical isolated footing is assumed based on the design principles outlined in IS 6403 [29] and is shown in Figure 12.

In Stage I, the embodied carbon emissions from the materials used in the stabilization of soil beneath an isolated footing are calculated based on data from Hammond and Jones [30,31].

The embodied carbon emissions from soil, gelatin, and water are estimated using carbon intensity values from the referenced sources [30,31].

The results, including total embodied carbon emissions for each material and overall, are presented in Table 2.

In Stage II, the embodied carbon emissions from the procurement and transportation of materials are assessed. Material extraction is carried out using a pickup excavator with a capacity of 10 t/L, while a heavy-duty dumper with a 25 t/L capacity is used to haul the materials over a distance of 1 km. The ECF for this stage is determined by the type of fuel powering the equipment, with values sourced from [32,33,34]. Table 3 presents the total embodied carbon emissions resulting from both procurement and haulage, taking into account the vehicle type and capacity, number of trips, fuel type, and haulage distance. Although the carbon intensity of the materials remains constant, the haulage distance significantly influences the total embodied carbon associated with this operation.

In Stage III, the total embodied carbon emissions generated during site operations are estimated. These operations include spreading the soil, thoroughly mixing it with gelatin at 1% by weight using a mechanical mixer, and compacting the stabilized soil with a smooth wheel roller. The carbon emissions from the equipment used in each of these activities are detailed in Table 4.

A summary of the embodied carbon emissions in the three phases is collectively represented in Table 5.

To assess the sustainability of gelatin-stabilized soil beneath the isolated footing, a comparison was made with conventional chemical stabilizers such as cement and lime. For the carbon emission analysis, dosages of 4% for cement and 6% for lime were used, based on values reported by Prusinski and Bhattacharja [35,36]. This analysis focused on Phase 1 to clearly understand the contributions of different materials to overall carbon emissions. Excessive use of cement and lime not only accelerates global warming but also negatively affects soil quality and local ecosystems due to increased pH levels [11]. Cement production alone emits approximately 1 ton of CO_2_ per ton of material [37], highlighting the need for alternative materials with lower carbon footprints [38]. In the present study, gelatin was used as a sustainable stabilizer at 1% by weight of soil, and the results showed a significant reduction in carbon emissions compared to traditional stabilizers. Specifically, cement and lime contributed 2207 CO_2_/t and 2545.3 CO_2_/t of the total emissions, respectively, while gelatin accounted for only 0.051 CO_2_/t as illustrated in Table 6. The embodied carbon factors (ECF) considered for cement and lime were 0.95 and 0.76, respectively [30,31,39]. The results show that the carbon emissions were reduced by 99.8% and 99% compared to traditional stabilizers such as lime and cement. Adding gelatin reduces costs and offers an environmentally friendly alternative to conventional stabilizers, especially in areas prone to harsh climatic conditions.

## 5. Conclusions

Gelatin was shown to be successful in stabilizing low-compressible soil, as demonstrated by a characterization test of gelatin. The gelatin-stabilized soil liquid limit and plastic limit increased by 76% and 56%, respectively, as compared to unamended soil at a maximum dosage of 2% gelatin. The MDUW of treated kaolin decreased by 12.7% at 2%. The UCS test revealed a significant increase with the 1% gelatin dosage increased by 1.69 times after a 1-day curing period. However, the same dosage experienced a significant increase by 4.25 times modulus during the 28-day curing period when compared to untreated soil. This increase was due to the presence of hydroxyl functional groups (-OH) and amide groups in the gelatin molecular structure. These groups can form hydrogen bonds with water molecules and other components in the soil, such as clay particles or silts. The hydrogen bonding not only stabilizes the soil particles but also helps in reducing soil plasticity and increasing stiffness. XRD results showed the formation of cementitious materials, such as CSH (Calcium Silicate Hydrate) and CaCO_3_ (Calcium Carbonate), suggesting the chemical stabilization of the soil. SEM analysis shows particle aggregation and the development of a gel-like structure that enhances soil cohesion, stability, and stiffness, the potential of gelatin as a stabilizing agent for improving the geotechnical properties of low-compressible soils. These results underscore the promise of using gelatin as an environmentally friendly and cost-effective alternative for soil stabilization. Overall, the study demonstrates that gelatin can be a highly effective and sustainable solution for enhancing the stability and strength of soils, particularly those with low compressibility. The development of a gel-like structure through the incorporation of gelatin not only improves soil cohesion but also significantly increases its strength, as demonstrated by the UCS test results. The environmentally friendly and cost-effective nature of gelatin as a stabilizing agent further solidifies its potential as a viable option for improving geotechnical properties in various construction and engineering projects. Additionally, the use of gelatin can significantly lower global warming potential by reducing carbon emissions, offering a more sustainable alternative to the high carbon footprint associated with cement and lime. The biodegradability of gelatin makes it a more sustainable option compared to traditional stabilizers. This means that once the construction project is complete, the soil can naturally break down the gelatin without causing harm to the environment. Overall, the use of gelatin in soil stabilization showcases its versatility and effectiveness in enhancing the overall performance of soil.

## Figures and Tables

**Table 1:** Geotechnical properties of soil.

Geotechnical Properties	Value
Liquid limit (%)	25
Plastic limit (%)	18.28
Plasticity index (%)	6.72
Specific gravity	2.71
Maximum dry unit weight (MDUW) (kN/m^3^)	19.23
Optimum moisture content (OMC) (%)	10

**Table 2:** Evaluation of carbon emissions from materials.

Stage I	Materials (1)	Amount (m^3^) (2)	Unit Weight (t/m^3^) (3)	Weight (t) (4)	ECF (5)	CO_2_ e/t (6) = (4)*(5)
Embodied carbon of material	Soil	3.33	1.92	6.39	0.02	0.127
	Gelatin	0.037	1.74	0.064	10.5	0.67
	Water	1.03	1	1.03	0.0010	1.03 × 10^−3^
**Total CO_2_ (t) emissions in Stage I = 0.79**						

ECF—embodied carbon factor.

**Table 3:** Evaluation of carbon emissions from procurement and haulage.

Stage II		Process	Vehicle	Capacity (t/L)	No of Loadings	Fuel (L)	ECF	CO_2_ e/t
Excavation and procurement		Soil procurement	Pickup excavator	10	0.639	0.639	3.25	2.07
		Gelatin procurement	Pickup excavator	10	0.0064	0.0064	3.25	0.020
**2.09**								
**Stage II**	**Process**	**Vehicle**	**Capacity** **(t/L)**	**Distance (km)**	**Trips**	**Fuel (L)**	**ECF**	**CO_2_ e/t**
Haulage	Soil	Heavy-duty dumper	25	1	1	0.31	3.25	1.007
	Gelatin	Heavy-duty dumper	25	1	1	1.28 × 10^−3^	3.25	0.00416
**Total CO_2_(t) emissions in Stage II = 3.54 1.011**								

**Table 4:** Evaluation of carbon emissions from site operations.

Stage III	Process	Vehicle	Capacity (t/L)	Trips	Fuel (L)	ECF	CO_2_ e/t
Site operations	Spreading	Bull dozers	10	0.63	0.63	3.25	2.04
	Mixing	Slurry mixer	0.5	14.9	14.9	3.25	48.42
	Compaction	Smooth wheel roller	12	0.623	0.623	3.25	2.02
**Total CO_2_(t) emissions in Stage III = 52.48**							

**Table 5:** Embodied carbon emissions from the three phases.

Stage	Operation	Embodied Carbon (CO_2_ e/t)
Stage I	Materials	0.79
Stage II	Procurement and haulage	3.54
Stage III	Site operations	52.48
**Total CO_2_(t) emissions = 56.81**		

**Table 6:** Embodied carbon emission comparison of gelatin with lime and cement.

Material	Dosage (%)	Quantity Required (t)	ECF	Embodied Carbon (CO_2_ e/t)
Gelatin	1	0.064	0.8	0.051
Lime	6	3349.17	0.76	2545.3
Cement	4	2232.78	0.95	2121.14

## Data Availability

All the data relevant to the manuscript are presented in the manuscript.
